# Mechanisms of Cytokine Modulation and Inflammatory Cascade in Bio-stimulatory Skin Rejuvenation: A Comprehensive Literature Review of Poly-L-Lactic Acid, Calcium Hydroxyapatite, Polycaprolactone, and Poly-D, L-Lactic Acid

**DOI:** 10.7759/cureus.107800

**Published:** 2026-04-27

**Authors:** Julio César Flores Rodríguez, Frank Eduardo Rosengaus Leizgold, Nathania Cárdenas Sicilia, Brenda Mariel Porras Zamora, Rodrigo Merino Arellano, Nuria Montserrat Rico Macías, Samara Susana Cabello Martínez

**Affiliations:** 1 Aesthetic and Regenerative Medicine, Clínica Aura, Monterrey, MEX; 2 Aesthetic and Regenerative Medicine, Sociedad Mexicana de Investigación en Medicina Estética (SOMIME), Monterrey, MEX; 3 Aesthetic and Facial Plastic Surgery, Ultimate Medica, Mexico City, MEX; 4 Aesthetic and Regenerative Medicine, The Youth Conspiracy, Mexico City, MEX; 5 Aesthetic and Regenerative Medicine, Consultorio Particular Dra. Brenda Mariel Porras Zamora, Mexico City, MEX; 6 Aesthetic and Regenerative Medicine, Sociedad Mexicana de Investigación en Medicina Estética (SOMIME), Mexico City, MEX; 7 Plastic Surgery, TecSalud, San Pedro Garza García, MEX; 8 Aesthetic and Regenerative Medicine, Clínica Nuria Rico, Tijuana, MEX; 9 Aesthetic and Regenerative Medicine, Sociedad Mexicana de Investigación en Medicina Estética (SOMIME), Tijuana, MEX; 10 Aesthetic, Regenerative, and Anti-Aging Medicine, Universidad Autónoma de Nuevo León, Monterrey, MEX; 11 Aesthetic, Regenerative, and Anti-Aging Medicine, Sociedad Mexicana de Investigación en Medicina Estética (SOMIME), Monterrey, MEX

**Keywords:** biostimulatory fillers, calcium hydroxyapatite (caha), cytokine modulation, inflammatory cascade, l-lactic acid (pdlla), polycaprolactone (pcl), poly-d, poly-l-lactic acid (plla)

## Abstract

Biostimulatory fillers, such as poly-L-lactic acid (PLLA), poly-D,L-lactic acid (PDLLA), polycaprolactone (PCL), and calcium hydroxyapatite (CaHA), have transformed aesthetics by promoting neocollagenesis through regulated inflammatory and cytokine-driven cascades. Their clinical effectiveness is well-established, but there is a lack of a synthesis of their mechanisms of action. The proposed comprehensive narrative literature review synthesized the contemporary evidence on the cytokine-modulating mechanisms and pathways of inflammatory responses induced by PLLA, PDLLA, PCL, and CaHA. The study design is a narrative literature review, which is performed using a systematic approach based on PRISMA 2020 guidelines. As a comprehensive literature review incorporating heterogeneous and predominantly non-comparative study designs, a formal risk-of-bias assessment using standardized tools was not performed; however, study limitations were considered qualitatively. It matched the identified mechanistic processes with clinical outcomes and safety profiles. The review included 22 clinical studies published from 1stJanuary 2010 to 28th February 2026, comprising randomized controlled trials, cohort studies, and quasi-experimental designs. Extracted data were systematically categorized into six predefined domains: acute inflammatory profile, macrophage polarization, fibroblast extracellular matrix (ECM) response, angiogenesis, clinical outcomes, and adverse events based on thematic analysis of study endpoints. All four biostimulators elicit a controlled inflammatory response characterized by macrophage infiltration and, in some cases, foreign-body giant cell formation. PLLA and PDLLA promote a transition from pro-inflammatory to pro-regenerative M2 macrophage phenotypes, driving sustained upregulation of Type I and III collagen, elastin, and angiogenesis. PCL induces durable neocollagenesis lasting up to 24 months, supported by neovascularization. CaHA stimulates fibroblast activity and ECM production, with a more pronounced early inflammatory gene signature than PLLA. Clinically, all biostimulators achieve significant and sustained improvements in wrinkle severity, skin quality, and volume restoration, with high patient satisfaction rates. Adverse events were predominantly mild and transient, with nodule formation being most notable with PLLA (4.7-28.6%). The study concluded that PLLA, PDLLA, PCL, and CaHA each elicit distinct yet overlapping inflammatory and cytokine-mediated cascades that culminate in robust neocollagenesis and tissue remodeling. Understanding these mechanistic differences enables clinicians to tailor treatment selection and protocols to achieve optimal, durable aesthetic outcomes with favorable safety profiles

## Introduction and background

Aesthetic rejuvenation has undergone a significant paradigm shift over the past decade, moving from the passive replacement of lost volume toward the active regeneration of tissue architecture [1]. This transition has been largely driven by the development of biostimulatory fillers, a class of injectable biomaterials that harness the body’s natural wound-healing processes to achieve long-term structural and functional skin improvement [2]. Unlike conventional hyaluronic acid (HA) fillers, which provide immediate but transient volumization through water-binding effects, biostimulatory agents including poly-L-lactic acid (PLLA), poly-D,L-lactic acid (PDLLA), polycaprolactone (PCL), and calcium hydroxyapatite (CaHA) function as temporary scaffolds that gradually stimulate endogenous tissue remodeling [3]. At the biological level, these agents promote dermal regeneration through activation of fibroblasts, the primary cells responsible for producing extracellular matrix (ECM) components such as collagen and elastin. This process is regulated by cytokine signaling, which refers to the coordinated communication between immune and structural cells via soluble molecular mediators. Key cytokines involved include transforming growth factor-β (TGF-β), which promotes collagen synthesis and tissue repair, and interleukin-10 (IL-10), which modulates inflammation and supports a regenerative healing environment [4]. The overall result is progressive neocollagenesis and dermal thickening, leading to gradual, natural-appearing improvements in skin quality and volume that may persist long after the material itself has been resorbed [5].

The regenerative effects of biostimulatory fillers are closely linked to the controlled inflammatory response they induce following injection [6]. Once introduced into the dermis, their particulate nature is recognized by the innate immune system as a foreign material, initiating a localized wound-healing cascade. This begins with an acute inflammatory phase characterized by recruitment of neutrophils and monocytes. Monocytes subsequently differentiate into macrophages, which are key regulatory cells in tissue repair and extracellular matrix remodeling, the process by which old or damaged matrix components are degraded and replaced with newly synthesized structural proteins [7]. Macrophages may attempt to phagocytose the injected particles; however, when the biomaterial exceeds a certain size threshold, complete engulfment is not possible. This leads to the fusion of macrophages into multinucleated foreign body giant cells (FBGCs), a hallmark of the foreign body reaction [8]. Importantly, macrophages can adopt different functional states, a phenomenon known as macrophage polarization. Pro-inflammatory (M1-like) macrophages dominate early, contributing to initial signaling and debris clearance, while a shift toward pro-repair (M2-like) macrophages supports tissue regeneration, fibroblast activation, and collagen deposition. The balance and timing of this transition, shaped by material properties such as composition, particle size, and degradation kinetics, are critical determinants of the magnitude and quality of the regenerative response [9].

The purpose of this review is to present a synthesis of the available evidence on the differential mechanisms of action of the four most widely used biostimulatory agents: PLLA, PDLLA, PCL, and CaHA, with particular emphasis on their modulation of the inflammatory cascade. This review investigates how each biomaterial interacts with the immune system in a distinct and material-specific manner, beginning with the acute inflammatory response and macrophage polarization kinetics and extending to downstream effects on fibroblast activation, ECM remodeling, and angiogenesis. These complex and finely regulated biological interactions are central to the regenerative potential of these agents. A deeper understanding of these processes is of paramount importance to clinicians, as it enables optimization of treatment protocols, informed selection of the most appropriate biostimulator for individual patient needs, and ultimately, maximization of the regenerative outcomes achievable with this class of aesthetic therapeutics.

## Review

Methodology

The study design is a narrative literature review, which is performed using a systematic approach based on Preferred Reporting Items for Systematic Reviews and Meta-Analyses (PRISMA) 2020 guidelines, except for risk of bias, quality assessment, certainty of evidence, and statistical quantitative synthesis. An extensive literature search was performed in PubMed, Cochrane, and Google Scholar databases from 1st January 2010 to 28th February 2026. The search strategy incorporated combinations of keywords such as Poly-L-lactic acid, PLLA; Poly-D,L-lactic acid, PDLLA; Polycaprolactone, PCL; Calcium Hydroxyapatite, CaHA; biostimulator; collagen stimulation; inflammation; cytokine; macrophage polarization; and neocollagenesis (Table 1). The inclusion criteria were (1) original clinical trials conducted in human subjects; (2) studies evaluating PLLA, PDLLA, PCL, or CaHA as an intervention in aesthetic indicators, and (3) studies reporting at least one outcome that addressed inflammatory response, tissue remodeling, clinical efficacy, and safety outcomes. The exclusion criteria included animal research, non-clinical in vitro research, and non-English articles.

**Table 1:** Search string

Database	String
PubMed	("cytokine"[Title/Abstract] OR cytokines[Title/Abstract] OR inflammation[Title/Abstract] OR "immune modulation" [Title/Abstract] OR "inflammatory response" [Title/Abstract] OR "immune response" [Title/Abstract] OR "molecular mechanism"[Title/Abstract]) ) AND ( ("biostimulatory" [Title/Abstract] OR "skin rejuvenation" [Title/Abstract] OR "regenerative skin" [Title/Abstract] OR "dermal regeneration" [Title/Abstract] OR PLLA OR "polylactic acid" OR CaHA OR "calcium hydroxyapatite" OR PCL OR "polycaprolactone" OR PDLLA OR "poly-D,L-lactic acid" OR "biostimulatory filler.") OR "collagen stimulation") AND (humans [Mesh] OR humans [All Fields])
Google Scholar	("cytokine modulation" OR "inflammatory cascade") AND ("biostimulatory filler" OR "skin rejuvenation") AND (PLLA OR CaHA OR PCL OR PDLLA OR "polylactic acid") AND (mechanism OR molecular OR fibroblast)
Cochrane Library	(cytokine OR inflammation OR immune) AND (skin OR dermal OR aesthetic OR rejuvenation) AND (plla OR caHa OR pcl OR pdlla OR filler)

Data were systematically compiled into a uniform table, including author and year, study design, sample size, type of biomaterial, treatment regimen, and follow-up period. Regarding mechanistic analysis, six outcome domains, i.e., (1) acute inflammatory profile (histological and clinical evidence of inflammation); (2) macrophage polarization (phenotypic markers and cytokine expression); (3) fibroblast ECM response (collagen upregulation, dermal thickening, and histological changes); (4) angiogenesis (evidence of neovascularization); (5) clinical outcomes (validated scales, patient satisfaction, and objective measurements); and (6) adverse events (type), were considered. A narrative synthesis strategy was used to combine the results obtained in biomaterials and explain the similar and different mechanistic pathways, given the diversity in study designs, outcome measures, and follow-up periods.

Selection process of studies

**Figure 1:**
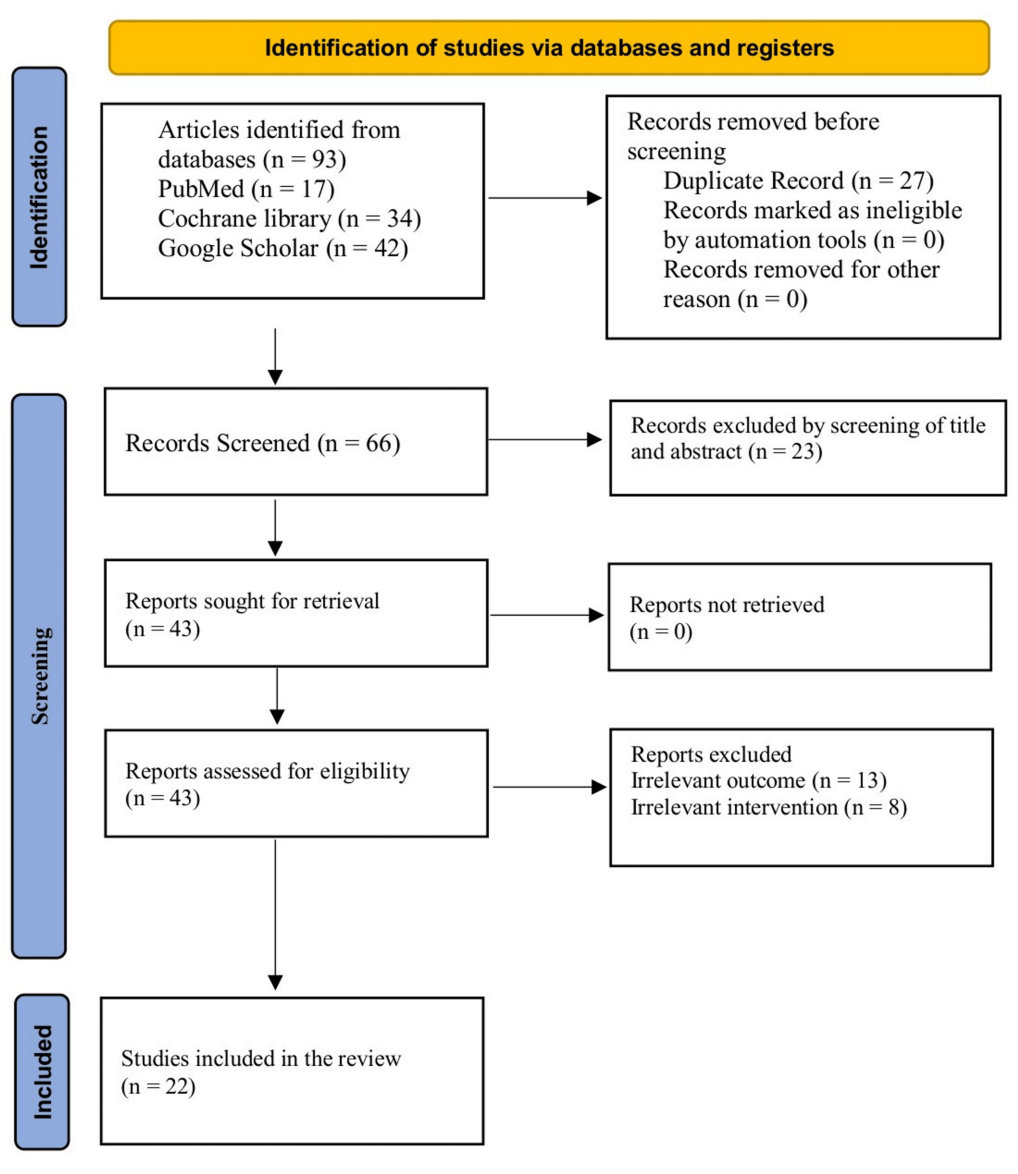
PRISMA flow chart PRISMA: Preferred Reporting Items for Systematic Reviews and Meta-Analyses

The initial database query provided 93 records, including 17 PubMed records, 34 Cochrane Library records, and 42 Google Scholar records. Twenty-seven duplicate records were removed prior to the screening phase. The remaining 66 unique records were screened for title and abstract, resulting in the removal of 23 articles that were evidently unrelated to the research question. The remaining 43 reports were also searched and located with success. Then these 43 reports were evaluated in detail against the eligibility criteria. After this comprehensive review of the full text, 21 reports were also eliminated based on 13 irrelevant outcomes and eight irrelevant interventions. Finally, a total of 22 studies were incorporated in the final qualitative synthesis after meeting all the inclusion criteria (Figure 1).

The review has considered 22 clinical trials that underpin the evidence base for the inflammatory and remodeling responses to common biostimulators. The review includes 12 randomized controlled trials (RCTs), which provide the highest-quality data on comparative efficacy and safety, along with eight cohort studies, one (1) quasi-experimental trial, and one (1) observational study. Although the major outputs of these studies were mostly clinical (e.g., wrinkle severity, aesthetic improvement), their different designs and long-term follow-ups provide essential information regarding the longevity and stability of the tissue response to each biomaterial. The sample sizes are small mechanistic groups of 4-5 individuals and huge RCTs including more than 300 participants, which are the extremes between exploratory studies of tissue response and large-scale validation of clinical findings (Table 2).

**Table 2:** Characteristics of studies included in the review PLLA: Poly-L-lactic acid; PDLLA: Poly-D,L-lactic acid; PCL: Polycaprolactone; CaHA: Calcium hydroxyapatite; HA: Hyaluronic acid; NLF: Nasolabial folds.

Author	Study Design	Sample Size	Biomaterial	Treatment Protocol	Follow-up
Stein et al., (2015) [10]	Randomized controlled trial	21 healthy postmenopausal women	Poly-L-lactic acid (PLLA)	Subcutaneous injections to both ventral upper arms: a total of 1200 mg per volunteer (600 mg/arm) in four sessions at three-month intervals; each 150 mg vial diluted in 8 mL water and 2 mL lidocaine 2%	Up to 28 months post-last injection
Zhang et al., (2025) [11]	Randomized controlled trial	331 (PLLA: 166, HA: 165)	Poly-L-lactic acid (PLLA) vs. hyaluronic acid (HA; Restylane Perlane Lidocaine)	PLLA: ≤3 sessions (four weeks apart), reconstituted with 5 mL saline; HA: ≤2 sessions; injected via bolus and fanning techniques	12 months
Bohnert et al., (2019) [12]	Randomized controlled trial	40 enrolled; 33 analyzed	Poly-L-lactic acid (PLLA) (Sculptra Aesthetic)	Three treatments (four weeks apart); reconstituted to 9 mL; injected into deep dermis using the tunneling technique	12 months post-last treatment
Narins et al., (2010) [13]	Randomized controlled trial	233 (116 PLLA, 117 collagen)	Poly-L-lactic acid (PLLA) vs human collagen	Injections into nasolabial folds (NLF) every three weeks (≤4 sessions); PLLA reconstituted with 5 mL sterile water	Up to 25 months
Srituravanich & Rummaneethorn, (2025) [14]	Quasi-experimental study	15	Poly-D,L-lactic acid (PDLLA) (AestheFill)	Two sessions (two months apart); vial reconstituted with 10 mL; injected via 23G cannula	6 months
Urdiales‐Gálvez et al., (2026) [15]	Randomized controlled trial	36 enrolled; 32 analyzed	Poly-L-lactic acid (PLLA) (PLLA-LASYNPRO, Julaine)	≤3 administrations (baseline, week 4, week 8); injected using the fanning technique	6 months
Seo et al. (2024) [16].	Randomized controlled trial	16 (15 F, 1 M)	Poly-D,L-lactic acid (PDLLA) + HA (Juvelook)	Intradermal mesotherapy; 2–3 sessions (4-week intervals); diluted to 10 mL	3–4 months
Petrone et al., (2025) [17]	Cohort study	32	Calcium hydroxyapatite (CaHA)	Single session, diluted (1:1), injected via a cannula in the fan pattern	12 months
Wan et al., (2025) [18]	Cohort study	4	Poly-D,L-lactic acid (PDLLA) (Juvelook)	Single session: tear trough injection using a cannula	6 months
Schierle & Casas (2011) [19]	Cohort study	106	Poly-L-lactic acid (PLLA) (Sculptra Aesthetic)	Panfacial staged injections using grid technique, reconstituted with lidocaine	≥24 months
Wan, Seo, et al., (2025) [20]	Cohort study	15	Poly-D,L-lactic acid (PDLLA) (Juvelook)	Three sessions (three weeks apart); microneedling + topical PDLLA	~5.5 months
Seo, Wan, et al., (2024) [21]	Cohort study	5	Poly-D,L-lactic acid (PDLLA) + HA (Juvelook)	Five sessions; needle-free injector delivery	5.5 months
Ponzo et al. (2025) [22].	Observational study	30	Polycaprolactone (PCL)	Two sessions (30 days apart); deep dermal injection	6 months
Zhao et al., (2023) [23]	Randomized controlled trial	160 (80 PCL, 80 HA)	Polycaprolactone (PCL) vs HA	Single injection into NLF	12 months
Byeon et al., (2026) [24]	Cohort study	7	Polycaprolactone (PCL) (GOURI®)	Single session; cannula-based injection	Up to 3–7 months
Moers‐Carpi & Sherwood (2013) [25]	Randomized controlled trial	40	Polycaprolactone (PCL) (Ellansé-S, Ellansé-M)	Initial + 1-month touch-up injection	24 months
Angelo-Khattar (2022) [26]	Cohort study	9	Polycaprolactone (PCL) (Ellansé-S)	Single session; supraperiosteal injection	24 months
Hong & Park, (2025) [27]	Cohort study	15	Calcium hydroxyapatite (CaHA) (Volassom)	Single session; subdermal injection	6 months
Park, (2025) [28]	Randomized controlled trial	10	Calcium hydroxyapatite (CaHA) (Volassom)	Single session; diluted CaHA injected at multiple points	1 month
Waibel et al., (2025) [29]	Randomized controlled trial	21	Poly-L-lactic acid (PLLA) vs CaHA	Split-face NLF injection; biopsy analysis	3 months
Pan et al., (2025) [30]	Randomized controlled trial	210	Calcium hydroxyapatite (CaHA) vs HA	Single split-face NLF injection	12 months
Arruda et al., (2024) [31]	Randomized controlled trial	10	Poly-L-lactic acid (PLLA) vs saline	Three sessions (four-week intervals)	4.5 months

PLLA is the most commonly described biostimulator in this list, as the primary intervention in nine studies [10-13,15,19, 29-31]. The investigations record its use in sites such as the nasolabial folds and panfacial, with follow-up of up to 28 months [10], providing a longitudinal perspective on the chronic inflammatory and fibroplastic processes it provokes. The more recent literature (2024-2026) shows a marked shift toward PDLLA [14,16,18,20,21] and PCL [22-26], typically in direct competition with HA or CaHA [17,27-30].

The fact that treatment interventions across the studies are heterogeneous is an important factor to consider in interpreting the resulting inflammatory stimulus. Some of these protocols used a single injection session to induce a controlled pro-inflammatory response [17,18,23,26-28,30], whereas others used progressive treatments over months [10-15,20-22,25], which might work by intensifying or extending the stipulated pro-inflammatory signaling pathways through cytokines. Moreover, other variables, such as reconstitution (e.g., volume of dilution, inclusion of lidocaine), depth of injection (deep dermis to supra-periosteal), and technique (cannula versus needle, bolus versus fanning), are important variables that determine the dispersion of the material, the area of the surface of the resultant inflammatory interface, and eventually the extent and quality of the resultant neocollagenesis.

Importantly, the follow-up times used in these studies are sufficiently long to track the chronology of the inflammatory and remodeling cascade. Most studies reported a 6-12-month [11,18,20,22,24,27,29-31] follow-up, which reflects the acute inflammatory reaction, the subsequent proliferative phase of fibroblast stimulation, and the early development of new extracellular matrix. This mechanistic review is facilitated by the addition of longer-term data out to 24 months in PCL and CaHA [25,26] and 28 months in PLLA [10,13,19] because it will provide insight into the resolution phase of inflammation and the long-term remodeling and stability of the newly synthesized collagen, distinguishing between these dynamic biostimulators and inert temporary volume fillers (Table 3).

**Table 3:** Summary of findings included in the study PLLA: Poly-L-lactic acid; PDLLA: Poly-D,L-lactic acid; PCL: Polycaprolactone; CaHA: Calcium hydroxyapatite; HA: Hyaluronic acid; ECM: Extracellular matrix; TGF-β1: Transforming growth factor beta 1; Tissue inhibitor of metalloproteinase 1; PDGFB: Platelet-derived growth factor subunit B; α-SMA: Alpha-smooth muscle actin; FBGCs: Foreign body giant cells; mRNA: Messenger RNA; TEWL: Transepidermal water loss; WSRS: Wrinkle severity rating scale; GAIS: Global aesthetic improvement scale; MFVDS: Midface volume deficit scale; IGA: Investigator’s global assessment scale; AEs: Adverse events; SAEs: Serious adverse events

Author	Acute Inflammatory Profile	Macrophage Polarization	Fibroblast ECM Response	Angiogenesis	Clinical Outcomes	Adverse Events
Stein et al., (2015) [10]	Infiltration of CD68⁺ macrophages and lymphocytes; formation of foreign body giant cells (FBGCs) around PLLA particles	Transition from early M2a (PDGFB-expressing) to late M2c phenotype (high TGF-β1 production)	Upregulation of Col I (3.25×) and Col III (0.43×) mRNA; increased TGF-β1 (0.19×) and TIMP1 (0.77×); histology confirmed Col III deposition around PLLA and Col I at capsule periphery; CD90⁺ fibroblasts and α-SMA⁺ myofibroblasts present	Increased α-SMA⁺ endothelial cells indicating neovascularization	Supports biological basis of volumizing effect	Nodules in 28.6%; foreign body reaction observed in all biopsies
Zhang et al., (2025) [11]	PLLA degradation produces lactic acid, stimulating fibroblasts/macrophages but may induce foreign body reactions	PLLA-SCA enhances anti-inflammatory pathways and adipocyte regeneration (distinct from CaHA)	Promotes collagen synthesis and tissue remodeling	Increased angiogenesis histologically	MMVS (12 mo): PLLA 90.57% vs HA 51.01% (p < 0.05); higher GAIS and satisfaction	AE incidence: PLLA 77.11% vs HA 70.12%; nodules 10.2%; mostly mild and transient
Bohnert et al., (2019) [12]	Foreign body reaction with inflammatory response resolving by 6 months	Not assessed	Formation of vascularized connective tissue; increased dermal thickness up to 2 years	Indirect evidence via vascularized tissue formation	Improved radiance, elasticity, hydration	None reported
Narins et al., (2010) [13]	Mild, transient injection-site reactions	Not assessed	Gradual ECM stimulation; peak correction at ~192.7 days	Not assessed	Significant WAS improvement vs collagen (P < 0.001)	PLLA-related AEs: 20.7% (early), 2.8% (long-term); nodules: 6.9%
Srituravanich & Rummaneethorn, (2025) [14]	PDLLA induces controlled inflammatory response	Not assessed	Early hydration effect; later collagen-driven improvements (≥2 months)	Not assessed	Significant improvement in elasticity, TEWL, wrinkles (p < 0.001); satisfaction 100%	Mild erythema (6.67%)
Urdiales‐Gálvez et al., (2026) [15]	Mild inflammation peaking at 61.1% (week 1), resolving by 3 months	Non-inflammatory bioinductive mechanism vs traditional PLLA	Increased P1CP (type I collagen biomarker); improved dermal density and neocollagenesis	Not assessed	WSRS improvement ≥1 point in ~70%; high GAIS satisfaction	Mild, self-limiting reactions; no serious AEs
Seo, Park, et al., (2024) [16]	Mild procedural pain and erythema	PDLLA promotes M2 polarization and IL-10 expression	Increased collagen and elastin fibers; improved wrinkles and pigmentation	PDLLA-induced angiogenesis	Significant improvement in all aging parameters (p < 0.05)	No serious AEs; transient erythema (25%)
Petrone et al. (2025) [17]	Mild, transient local reactions	Not assessed	Increased dermal thickness (+51%), indicating ECM remodeling	Not assessed	75% improvement (GAIS); improved laxity	Mild bruising, pain, edema (self-resolving)
Wan, Hidajat, et al., (2025) [18]	Mild, transient inflammation	PDLLA modulates macrophages (preclinical evidence)	Sustained collagen production	Not assessed	Significant tear trough improvement	No serious AEs
Schierle & Casas, (2011) [19]	PDLLA involves a localized inflammatory response	Macrophage recruitment	Collagen deposition and dermal fibroplasia	Not assessed	99.1% satisfaction	Nodules: 4.7%
Wan, Seo, et al., (2025) [20]	Micro-injury-induced inflammatory response (microneedling)	Not assessed	Neocollagenesis leading to pore reduction	Not assessed	Significant pore reduction (p < 0.001)	Mild erythema, edema
Seo, Wan, et al., (2024) [21]	Mild transient inflammation	PDLLA modulates macrophages	Promotes dermal collagen production	PDLLA stimulates angiogenesis	Improved GAIS and patient satisfaction	Mild, transient effects only
Ponzo et al. (2025) [22].	Mild-to-moderate inflammation (3.3%)	Not assessed	PCL stimulates collagen (Type III → Type I)	Not assessed	95% satisfaction; improved facial volume	Low AE rate (3.3%)
Zhao et al., (2023) [23]	Mild, transient reactions	Not assessed	Sustained ECM stimulation in PCL vs HA	Not assessed	WSRS improvement: PCL 88.8% vs HA 23.8% (p < 0.001)	No serious injection-related AEs
Byeon et al. (2026) [24]	Persistent bruising (atypical)	Not assessed	Dense scaffold formation	Not assessed	Complete resolution with treatment	No serious AEs
Moers‐carpi & Sherwood, (2013) [25]	Mild transient edema/ecchymosis	Macrophages resorb CMC carrier	Long-term neocollagenesis	Not assessed	Sustained WSRS/GAIS improvement	No major complications
Angelo-Khattar (2022) [26]	Inflammatory response with encapsulation	FBGC formation	Increased volume (50–150%); collagen I & III synthesis	Neovascularization observed	Significant volume enhancement	None reported
Hong & Park (2025) [27].	Mild transient inflammation	Not assessed	CaHA stimulates fibroblasts and ECM production	Not assessed	Improved MFVDS, elasticity, hydration	No serious AEs
Park (2025) [28]	Minimal inflammation	Not assessed	Increased collagen I/III, elastin	Not assessed	Improved TEWL, elasticity, wrinkles	Mild bruising only
Waibel et al. (2025) [29]	CaHA: pro-inflammatory gene expression; PLLA: lower inflammation	Not assessed	PLLA stimulated ECM regeneration; CaHA limited regenerative response	Not assessed	Not assessed	Not reported
Pan et al., (2025) [30]	Typical injection reactions (swelling, pain)	Not assessed	Indirect ECM stimulation	Not assessed	CaHA non-inferior to HA	Similar AE profiles
Arruda et al. (2024) [31]	No significant inflammation	Not assessed	Increased remodeling; reduced elastin fragmentation	Increased angiogenesis	Improved GAIS and skin quality	No treatment-related AEs

Acute inflammatory profile

An acute inflammatory response to biostimulators is defined as a localized, transient response that causes a cascade involving tissue remodeling. The analysis of the histological specimens of the tissue after the injection of PLLA shows a constant infiltration of CD68+ macrophages and lymphocytes and the development of FBGCs around the PLLA particles that correspond to the classic foreign body reaction [10]. The clinical manifestations of this inflammatory process are mild to moderate inflammation, edema, and erythema, which generally peak within the first week of treatment and heal spontaneously over days to months [12,15,16,18,20,21]. In the case of PCL-based fillers, the inflammatory response is also mild-to-moderate and self-limiting, and, in rare cases, facial swelling or periorbital edema may require medical intervention [22,25,26]. The presence of an inflammatory signature in CaHA-R is more pronounced than in PLLA-SCA, as evidenced by gene expression comparisons. Remarkably, certain contemporary formulations of PLLA are made to reduce this stage of inflammation, rather than induce a non-inflammatory, bioinductive response, which differs from the conventional systems of macrophage reaction [15]. The inflammatory profile is usually well-tolerated, and any adverse events are reversible and short-lived across all the biomaterials [11,14,17,23,27,28,30,31].

Macrophage polarization

The process of macrophage polarization is one of the determinants of tissue response to biostimulators, defining the transition between inflammation and tissue repair and regeneration. The macrophages experience a dynamic phenotypic shift after PLLA injection, initially an early-dominant M2a subtype capable of producing PDGFB and later a dominant M2c subtype capable of producing high levels of TGFb1, a transition that coordinates the fibroblastic response [10]. The existence of FBGCs also proves the presence of macrophages in the destruction and encapsulation of PLLA particles [10,26]. PDLLA has been demonstrated to enhance polarization toward M2 and IL-10 expression, thereby facilitating an anti-inflammatory, pro-regenerative microenvironment that promotes tissue remodelling [16]. PDLLA is an effective way to control macrophage behavior and stimulate collagen production [18,21]. In the case of PCL-based fillers, macrophages gradually resorb the carboxymethylcellulose (CMC) gel carrier, and FBGCs are observed to engulf the PCL microspheres [25,26]. The available studies provide limited mechanistic investigation, and their protocol descriptions and discussion of underlying mechanisms do not constitute explicit evaluation of macrophage polarization. Consequently, the role of macrophages as key mediators of the biostimulatory effects of PLLA, PDLLA, PCL, and CaHA remains poorly characterized [11,12,14,19,28]. Future studies should incorporate direct immunophenotyping approaches to address this gap, including immunohistochemical or flow cytometric assessment of macrophage subsets (e.g., CD68, CD80 for M1 phenotype and CD163, CD206 for M2 phenotype), alongside quantitative gene expression profiling and cytokine analysis to better define polarization dynamics.

Fibroblast ECM response

Fibroblast ECM response is the primary process in biostimulator-induced rejuvenation, leading to significant neocollagenesis and dermal remodeling. PLLA injection results in a significant increase in Col III and Col I mRNA, as well as TGFb1 and TIMP1. Histology reveals extensive deposition of Col III immediately surrounding PLLA particles and Col I at the periphery of the capsule, along with the presence of CD90+ fibroblasts and aSMA+ myofibroblasts [10]. This clinical manifestation is a progressive increase in dermal thickness and soft tissue volume that continues over the years [12,13,19]. PDLLA also stimulates collagen synthesis, and the early effects of skin hydration are due to immediate filling, with long-term effects beginning after two months due to neocollagenesis [5,7,9,11,12]. The histological studies conducted on PDLLA-treated skin reveal an increase in collagen and thickened elastic tissue fibers within the dermis [16]. PCL microspheres result in a persistent neocollagenic response; the degraded CMC gel carrier is substituted by new type I and III collagen, leading to stable aesthetic outcomes up to 24 months [23,25,26]. The effect of CaHA is on fibroblast activity and neocollagenesis, as improvements in skin elasticity, reduction in wrinkles, and dermal thickness were significantly enhanced by ultrasound and biophysical measurements [17,27,28,30]. Comparison studies indicate that PLLA-SCA activates more ECM factors associated with regenerative mechanisms than CaHA-R does [29].

Angiogenesis

**Figure 2:**
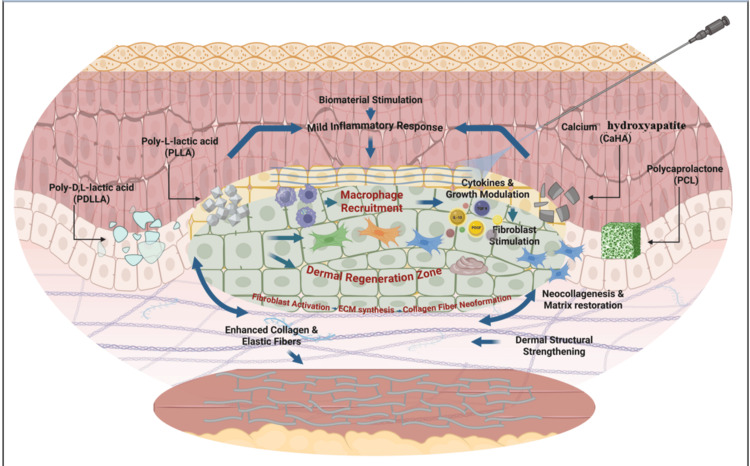
Schematic overview of biomaterial-host tissue interaction demonstrating controlled inflammatory signaling, cytokine modulation and dermal extracellular matrix regeneration induced by bio stimulatory injectable (PLLA, CaHA, PCL, PDLLA) Created in BioRender. Flores Rodríguez, J. C. (2026) https://BioRender.com/qo6h5nr

Angiogenesis plays a major role in tissue remodeling and helps the development of new, vascularized connective tissue after injection of a biostimulator. Treatment with PLLA leads to an increase in the number of aSMA+ endothelial cells differentiated into vessel-like structures in the granulation tissue, indicating active neovascularization [31]. This process of angiogenesis helps develop vascularized connective tissue, which facilitates long-term dermal thickening and enhances skin quality [12]. PDLLA is also projected to promote angiogenesis, which is another indication of its use in total tissue regeneration [16,21]. In the case of PCL-based fillers, new capillaries have been observed to form around the microspheres, and the perfusion of the newly produced extracellular matrix has been adequately ensured [26]. Although no studies have directly measured angiogenesis, the presence of vascularized granulation tissue and sustained tissue viability provides only indirect evidence; therefore, any inference of angiogenesis remains speculative and requires confirmation through direct quantitative assessment [11,13,17,23,25,27,30]. After reviewing the mechanism of action of cytokines, Figure 2 was created using BioRender to illustrate the mechanisms of action of biostimulatory injectables.

Adverse events

The adverse events related to biostimulator injections are mostly mild, temporary, and self-limiting and are manifestations of the anticipated tissue reaction to regulated inflammation and remodeling. Injection-site reactions, which encompass edema, pain, tenderness, erythema, and bruising, are the most prevalent in all biomaterials, and they usually resolve within hours to days [11,14,19,22,24]. A significant issue with PLLA is nodule formation, which occurs in 4.7 to 28.6 percent of patients, with most being mild and healing or improving over time [10,11,13,19]. PDLL and PCL exhibit lower nodule rates, and studies indicate no nodules or granulomas [20,22,25]. All biomaterials have few serious adverse events, and reported events are usually unrelated to the devices [11,23]. Long-term bruising that takes several months to heal after PCL injection has been reported in a limited number of cases, but all cases have been managed successfully [24]. In several studies, no serious adverse events were reported during the treatment process, which demonstrated the positive safety profile of these biostimulators when applied by trained practitioners [17,18,26,31].

Clinical efficacy

The clinical effectiveness of biostimulatory fillers has been well-established across all four classes of biomaterials, and it results in substantial and lasting improvements in aesthetic outcomes. PLLA has shown high response rates, and one large randomized controlled trial found an improvement in the Master Merz Visual Scale (MMVS) of 90.57 points, significantly better than hyaluronic acid (51.01, p < 0.05) [11]. The investigator's assessments of the Global Aesthetic Improvement Scale (GAIS) at 12 months showed similar improvement favoring PLLA (94.34% vs. 74.50%, p < 0.05), and subject satisfaction scores were significantly greater in PLLA [11]. The prolonged follow-ups (25-28 months) confirm the long-term wrinkle reduction and volume replacement achieved by PLLA after treatment [10,13,19]. PDLLA yields considerable changes in the various skin quality parameters at six months (elasticity, hydration, transepidermal water loss (TEWL), pore size, and wrinkles) (p < 0.001), and 93.3% of patients reported extreme satisfaction [14]. PDLLA alone, with microneedling or new delivery systems, exhibits long-term decreases in pore size and improvements in texture at 22 weeks without retreat [20,21]. The clinical results of all types of biostimulators show important, long-term positive changes in aesthetic parameters and patient satisfaction. PLLA has also demonstrated very high responder rates on the MMVS and GAIS, being statistically significantly better than hyaluronic acid at 12 months of treatment [11]. The 25-28-month follow-ups affirm continued wrinkle reduction and volume replacement [13,19]. PDLLA has been shown to make significant gains in elasticity, hydration, transepidermal water loss, pore size, and wrinkles after six months, with more than 93 percent of patients reporting satisfaction [14,16,18]. PCL fillers show outstanding durability, with 88.8% effectiveness at 12 months versus 23.8% in HA, and the process of continuous enhancement of mid-face volume (50-150% over baseline) has been observed to continue over 24 months [23,25,26]. At 24 weeks, CaHA achieves high success rates in midfacial volume deficiency, skin elasticity, barrier function, and hydration, with high patient satisfaction and GAIS results [17,27,28,30]. Across all biomaterials, patient satisfaction is very high, with satisfaction with PCL at 95 percent and with PLLA at 99.1 percent [19,22].The PCL fillers show high durability, with efficacy rates of 88.8% at 12 months and 100% of PCL-2 patients improving on GAIS at 24 months (p < 0.001) [23,25]. Sustained volume changes of 50-150 percent of the mid-face baseline were observed in 3D volumetric analysis in response to PCL injection two years later, and neocollagenesis directly correlates with ongoing neocollagenesis [26]. At 12 months, PCL still achieves very high patient satisfaction at 95 percent [22]. At 24 weeks, CaHA shows substantial increases in the volume deficiency of the midface; the midface volume mean score rises to 2.00 vs. 3.33 (p<0.001), as well as skin barrier functioning (reduced TEWL), hydration, and all measures of elasticity (p < 0.05) have improved [27,28]. In comparison, CaHA had a responder rate of 84.04 at week 24; it was non-inferior to HA [30]. Taken together, these clinical effects confirm the long-term, biostimulatory mechanism of action between these biomaterials, the effectiveness of which goes far beyond the degradation time of temporary fillers.

Discussion

The results of the current review indicate that the clinical effects of biostimulatory fillers, including PLLA, CaHA, PCL, and PDLLA, rely on a tightly controlled inflammatory cascade with initial cytokine activation and macrophages. The transition promotes fibroblast activation, ECM remodeling, and long-term neocollagenesis, and it couples these processes with angiogenesis. These combined mechanisms provide us a biological framework that explains the reduction of wrinkle severity, skin quality, and volume restoration.

The physicochemical analysis by Sedush et al. (2023) [32] highlights that PLLA/PDLLA filler compositions show a number of parameters (e.g., polymer composition, degree of crystallization, molecular weight distribution, and particle geometry) that can be expected to significantly affect the rates of hydrolytic degradation. Specifically, it has been observed that while PLLA is semi-crystalline and thus will likely undergo slower hydrolysis than amorphous PDLLA, PDLLA will exhibit more rapid hydrolysis due to its lack of ordered structure. However, these differences in the rate of hydrolysis between PLLA and PDLLA can be affected by other factors within the filler particles themselves, including whether they have porous versus nonporous morphologies. This is in line with the findings of the present review, specifically those of Zhang et al. (2025) [11] and Stein et al. (2015) [10], which demonstrated that PLA degradation into lactic acid could regulate fibroblast activation and macrophage behavior. The heterogeneous inflammatory reactions and adverse event patterns (e.g., nodules, foreign body reactions) on PLLA can be explained by variability in degradation kinetics. In this way, physicochemical heterogeneity serves as an important upstream factor that dictates downstream cytokine signaling and tissue remodeling.

Secondly, the preclinical macrophage study by Nowag et al. (2024) [33] provides solid support for the cytokine-mediated inflammatory variation between PLLA and CaHA. They show that PLLA substantially stimulates the expression of the pro-inflammatory cytokines IL-8, MIP-1α, and TNFRII, especially in M1 macrophages, and that CaHA produces a less inflammatory or regenerative effect. This aligns perfectly with the current review, which repeatedly associates PLLA with foreign body reactions, macrophage recruitment, and dynamic M2 polarization (M2a→M2c transition), while CaHA is defined by a less inflammatory, more bioinductive ECM remodeling profile. Notably, the recent results build on these by demonstrating that more recent PLLA formulations (e.g., PLLA-SCA) could shift toward a less inflammatory, regenerative phenotype, implying biomaterial design development to reduce the negative impact of cytokines [27,29].

Thirdly, the narrative review conducted by Aguilera et al. (2023) [34] provides additional supporting information regarding the regenerative paradigm of CaHA. The data from the present study confirm that CaHA functions as a primary scaffold for tissue engineering purposes and not just as an inert space-occupying material. Upon injection of CaHA into the body, it is possible that the microspheres of CaHA will have some degree of contact with nearby fibroblasts, thereby initiating various types of mechanical transduction within those cells, such as the activation of mechanoreceptors and cytoskeletal elongation. These forms of mechanical transduction can be expected to lead to a biosynthetic phenotype in which fibroblasts are recruited to sites adjacent to the injected CaHA, where they produce extracellular matrices and participate in tissue remodeling. In the context of this paradigm, CaHA has been referred to as a biostimulatory filler that induces fibroblast proliferation, stimulates collagen and elastin formation, promotes vascularization, and facilitates repair of damaged extracellular matrices without causing extensive fibrosis [34,35]. Recent clinical studies provide strong evidence supporting this model, as Park (2025) [28] and Hong & Park (2025) [27] report improved skin elasticity, hydration levels, and dermal thickness along with a relatively small number of patients who experienced inflammatory side effects. Overall, these results support the hypothesis that CaHA-induced tissue regeneration occurs through both the inherent scaffold properties of the material and biomechanical interactions between cells and materials that facilitate constructive tissue remodeling.

A study by Huth et al. (2024) [35] has shown that PLLA-SCA triggers the significant upregulation of pro-inflammatory and regulatory cytokines, including IL1B, TGFB2, and CXCL6, in the 3D skin models with macrophages. This study demonstrates that initial inflammatory signaling is not merely a transient reaction but a critical precursor to subsequent regenerative processes, particularly the synthesis of collagen I. This corresponds with the current review, which posits that cytokine modulation is an essential mechanism connecting inflammation to fibroblast-mediated tissue regeneration, thereby supporting the notion of a regulated, positive response to inflammation rather than an entirely negative one [36].

Waibel et al. (2024) [29] found that the expression levels of various genes in PLLA and CaHA differ. PLLA selectively stimulated ECM-related and regenerative signaling while maintaining a relatively lower level of inflammatory signaling, whereas CaHA showed an increase in pro-inflammatory-related genes. This deviation confirms our observation regarding the review that, despite the similarity of the endpoint, such as stimulation of collagen, the cytokine cascades and immune responses that initiate these responses in different biomaterials differ considerably. These differences could be the cause of variations in the onset of action, duration of results, and profile of adverse events; biomaterial-specific immunobiology is important [29].

In the preclinical trial conducted by Kwon et al. (2019) [37], PCL induced an orderly temporal response in tissues, as evidenced by inflammatory cell infiltrate at one week and then a significant reduction in inflammation from four weeks onward, without indication of severe adverse reactions to the foreign body or pathological fibrotic responses. This orderly process was transformed into a slow increase in type I collagen deposition, epidermis thickening, and the continuous increase in dermal collagen through time. The progression observed in these studies reflects that seen clinically in studies by Moers-Carpi and Sherwood [25], and Angelo-Khattar [26], where PCL-based products exhibited sustained neocollagenesis, long-term tissue remodeling, and long-term volumetric enhancement.

Zou et al. (2024) [38] demonstrated the metabolic aspect of the PLLA-stimulated regeneration. The discovery of lactate-mediated KAT8-dependent lactylation of LTBP1 as an inducer of collagen I and III production puts in place an epigenetic regulatory mechanism that supplements the cytokine-based pathways reported in our review. This observation broadens the knowledge base of biostimulation beyond the classical inflammatory signaling, indicating that bioproducts of biomaterials can directly regulate the expression of genes and protein synthesis in fibroblasts, which in turn leads to long-term tissue remodeling [38]. This helps verify the conclusion of this review: clinical efficacy is well-known, but researchers have not yet fully understood the mechanistic pathways of these biomaterials.

Limitations

Although the evidence strongly supports the effectiveness of the biostimulator, we must consider several limitations. There is a high level of heterogeneity in studies related to treatment protocols, as they vary in dilution volume, number of sessions, injection type (needle or cannula), injection depth, and anatomical location of the anatomy of interest. This diversity makes it difficult to compare biomaterials directly and to develop responsible, standardized, evidence-based treatment algorithms. The follow-up periods, though strong in other studies, are uneven, with some research relying on a 3-to-6-month follow-up, which could underestimate the potential course of neocollagenesis and late-onset adverse outcomes, such as nodules. The number of participants in mechanistic studies is typically small, which constrains the statistics and generalizability. The absence of standardized, validated outcome measures for skin quality parameters creates a risk of bias in evaluation.

Recommendations

To improve future studies, standardized treatment procedures are required for each class of biostimulators to make comparisons meaningful across studies. The priority should be given to mechanical studies that include serial biopsies, biomarker analysis (e.g., serum P1CP), and high-resolution imaging (ultrasound, 3D volumetric analysis) to enable direct correlation of the inflammatory cascade with clinical outcomes. Prospective studies that follow up beyond the 24-month mark must fully describe the resolution phase of inflammation and the sustainability of neocollagenesis. Trials that compare the various classes of biostimulator within the same group of patients would provide the strongest evidence for differences in efficacy and safety profiles. Outcome measures for skin quality parameters should be developed and standardized for consistent use. Since these biomaterials have immunomodulatory properties, their potential for use in the treatment of inflammatory skin disorders or as molecular-scale photoaging modulators warrants exploration.

## Conclusions

This review shows that injectable biostimulatory biomaterials share a common core pathway of inducing controlled foreign body reaction marked by macrophage recruitment and multinucleated giant cell formation that switches to a more profusively M2-polarized, regenerative microenvironment that stimulates fibroblast In spite of these observations, the literature is limited by heterogeneity of study design, small and uneven sample size, inconsistent follow-up times, non-standardized outcome measures, and a paucity of direct head-to-head comparisons and mechanistic studies that are able to combine histological, molecular, and imaging data, thus limiting translational clarity and generalizability. Future studies need to focus on well-powered randomized controlled studies using standardized protocols and long-term follow-up, as well as integrated clinical/preclinical frameworks that integrate molecular profiling, state-of-the-art imaging, and histopathological validation to clarify unexplained mechanistic pathways, define comparative biologic behavior using biomaterials, and support the creation of precision-based aesthetic therapies.
